# Data for Korean college students׳ anxious and avoidant attachment, self-compassion, anxiety and depression

**DOI:** 10.1016/j.dib.2017.06.006

**Published:** 2017-06-07

**Authors:** Ju Ri Joeng, Sherri L. Turner, Eun Young Kim, Seung Ae Choi, Jung Ki Kim, Yu Jeong Lee

**Affiliations:** a Department of Education, Chonnam National University, South Korea; b Counseling and Student Personnel Psychology, Department of Educational Psychology, University of Minnesota, Twin Cities, USA; c Department of Psychotherapy, Kyungil University, South Korea; d Counseling Center, Pohang University of Science and Technology, South Korea; e Counseling Center, POSCO, South Korea

## Abstract

The data presented in this article are from 473 Korean college students׳ responses to an online survey consisting of measures of anxious and avoidant attachment (the Experiences in Close Relationships-Revised Scale: ECR-R), self-compassion (Neff׳s Self-Compassion Scale: SCS), depression (the Center for Epidemiological Studies-Depression Scale: CES-D) and anxiety (the State-Trait Anxiety Inventory-the Trait Anxiety Scale: STAT-T). Each variable was measured by a Korean version of the instrument. Participants were recruited from three universities in South Korea: 288 were men and 185 were women; 199 were undergraduate and 273 were graduate students. The online program used to collect the data prompted for but did not require responses to items; 26 surveys were not completed, and data from these surveys were not included in the dataset. Major findings based on the data presented here are reported in the article “Insecure attachment and emotional distress: Fear of self-compassion and self-compassion as mediators” (Joeng et al., 2017) [1]. The data, an SPSS file, are included as supplementary material.

**Specifications Table**

**Table:** 

Subject area	*Psychology*
More specific subject area	*Insecure Attachment, Self-Compassion, Emotional Distress*
Type of data	*SPSS file*
How data was acquired	*Survey*
Data format	*Analyzed*
Experimental factors	*A cross-sectional research design*
Experimental features	*The variables were anxious attachment, avoidant attachment, self-compassion, depression, and anxiety.*
Data source location	*South Korea*
Data accessibility	*Data are provided as supplementary material*

**Value of the data**

• These data include information on insecure (anxious and avoidant) attachment, self-compassion, and emotional distress (anxiety and depression).
• These data were collected from Korean college students, which adds a dimension of cultural diversity to what is already known about the variables under study.
• These data could be used in meta-analyses to combine and compare estimates from different studies.

## 1 Data

The SPSS file provides the means for measures of anxious attachment, avoidant attachment, self-compassion (including for the subscales that are used to measure the dimensions of self-compassion: self-kindness, self-judgment, common humanity, isolation, mindfulness, and over-identification), depression, and anxiety from a sample of 473 Korean college students. Major findings based on the data presented here are reported in the article Insecure attachment and emotional distress: Fear of self-compassion and self-compassion as mediators (Joeng et al., 2017) [1].

## 2 Research design, materials, and methods

### 2.1 Participants

The data were collected from 473 students who attended university in South Korea; 288 (61%) male, 185 (39%) female; 199 (42%) undergraduate, and 274 (58%) graduate; mean age=25.26 years (*SD*=3.78). We recruited from three universities to maximize sample variability in terms of enrollment in diverse fields of study (one university offered degrees in the humanities and social sciences, one in the humanities, social sciences, and STEAM fields, and one in the science, technology, engineering, and mathematics fields). Participants were recruited through university email lists and websites, and from psychology classes. Participation was voluntary. Item responses were not required, but were encouraged. Responses from 26 incomplete surveys were not included in the data. Gender was coded 1 for male and 2 for female. Group was coded 1 for undergraduate and 2 for graduate.

### 2.2 Questionnaires

Anxious and avoidant attachment styles were measured using the Anxious (9 items) and Avoidant (9 items) Attachment subscales of the Experiences in Close Relationships-Revised Scale [2] (Korean version [3]). Items were measured on a 7-point Likert scale, with higher scores indicating greater anxious/avoidant attachment. Convergent validity was established between the English and Korean versions of the instrument via tests of factorial equivalency [3]. In this current dataset, Cronbach׳s α = .94 for Anxious Attachment and .79 for Avoidant Attachment.

Self-compassion was measured using the Self-Compassion Scale [4] (Korean version, [5]). This instrument is comprised of six subscales: Self-Kindness (5 items), Self-Judgment (5 items), Common Humanity (4 items), Isolation (4 items), Mindfulness (4 items), and Over-Identification (4 items). Each of the items was measured on a 5-point Likert Scale. For the Self-Kindness, Common Humanity, and Mindfulness subscales, greater scores indicated greater magnitude. For the Self-Judgment, Isolation, and Over-Identification subscales, greater scores indicated less magnitude. Self-compassion as measured by the Korean-version of SCS has been negatively related to depression and anxiety, and positively related to mental health, emotional regulation, and life satisfaction [5]. In this current dataset, Cronbach׳s α for Self-Compassion = .90.

Depression was measured using the 20-item Center for Epidemiological Studies-Depression Scale [6] (Korean version, [7]). Items were scored on a 4-point Likert scale, with higher scores indicating greater depression. Chon and colleagues [7] reported that similar construct validity was found when testing college students in Korea with the Korean version as when testing college students in the U.S. with the English version. In this current dataset, Cronbach׳s α for Depression = .92.

Anxiety was measured using the 20-item Trait Anxiety Scale of the State-Trait Anxiety Inventory [8] (Korean version, [9]). Items from this scale were rated on a 4-point Likert scale, with greater scores indicating more trait anxiety. Trait anxiety as measured by the Korean version of the Trait Anxiety Scale has good concurrent validity with trait anxiety as measured by the Taylor Manifest Anxiety Scale [10], [11]. In this current dataset, Cronbach׳s α for Trait Anxiety = .94.

### 2.3 Statistical analysis

Means of Means, SDs, and correlations among scale-derived variables are shown in Table 1.

**Table 1:** Descriptive statistics and correlations.

**Variables**	***X***	***SD***	**1**	**2**	**3**	3*a*	3*b*	3*c*	3*d*	3*e*	3*f*	**4**	**5**
1*.* Anxious attachment	3.86	.77	1.00⁎⁎⁎	.40⁎⁎⁎	−.42⁎⁎⁎	−.30⁎⁎⁎	−.29⁎⁎⁎	−.23⁎⁎⁎	−.37⁎⁎⁎	−.26⁎⁎⁎	−.25⁎⁎⁎	.40⁎⁎⁎	.41⁎⁎⁎
2*.* Avoidant attachment	3.05	1.04		1.00⁎⁎⁎	−.49⁎⁎⁎	−.10⁎	−.54⁎⁎⁎	−.10⁎	−.54⁎⁎⁎	−.15⁎⁎	−.51⁎⁎⁎	.50⁎⁎⁎	.50⁎⁎⁎
3. Self-compassion	3.14	.59			1.00⁎⁎⁎	.67⁎⁎⁎	.74⁎⁎⁎	.56⁎⁎⁎	.74⁎⁎⁎	.63⁎⁎⁎	.72⁎⁎⁎	−.59⁎⁎⁎	−.75⁎⁎⁎
3*a. Self-kindness*	2.64	.79				1.00⁎⁎⁎	.24⁎⁎⁎	.58⁎⁎⁎	.16⁎⁎⁎	.69⁎⁎⁎	.15⁎⁎⁎	−.27⁎⁎⁎	−.39⁎⁎⁎
3*b. Self-judgment*	2.48	.91					1.00⁎⁎⁎	.06	.71⁎⁎⁎	.10⁎	.75⁎⁎⁎	−.52⁎⁎⁎	−.65⁎⁎⁎
3*c. Common humanity*	2.87	.86						1.00⁎⁎⁎	.15⁎	.61⁎⁎⁎	.04	−.22⁎⁎⁎	−.27⁎⁎⁎
3*d. Isolation*	2.63	.96							1.00⁎⁎⁎	.16⁎⁎	.72⁎⁎⁎	−.60⁎⁎⁎	−.70⁎⁎⁎
3*e. Mindfulness*	2.96	.80								1.00⁎⁎⁎	.19⁎⁎⁎	−.26⁎⁎⁎	−.33⁎⁎⁎
3*f. Over-Identification*	2.30	.89									1.00⁎⁎⁎	−.49⁎⁎⁎	−.67⁎⁎⁎
4. Depression	1.89	.53										1.00⁎⁎⁎	.73⁎⁎⁎
5. Anxiety	2.17	.51											1.00⁎⁎⁎

*Note. X =* Mean of Means; 3*a* through 3*f* are the variables used to construct the Self-compassion variable.

⁎ *p*<.05

⁎⁎ *p*<.01

⁎⁎⁎ *p*<.001

## References

[1] Joeng J.R., Turner S.L., Eun Y.K., Seung A.E., Lee Y.J., Kim J.K. (2017). Insecure attachment and emotional distress: fear of self-compassion and self-compassion as mediators. Personal. Individ. Differ..

[2] Fraley R.C., Waller N.G., Brennan K.A. (2000). An item response theory analysis of self-report measures of adult attachment. J Personal. Soc. Psychol.

[3] Kim S.H. (2004). Adaptation of the Experiences in Close Relationships-revised Scale into Korean: Confirmatory Factor Analysis and Item Response Theory Approaches (Unpublished master׳s thesis).

[4] Neff K.D. (2003). The development and validation of a scale to measure self-compassion. Self Identity.

[5] Kim K., Yi G., Cho Y., Chai S.J., Lee W. (2008). The validation study of the Korean version of the self-compassion scale. Korean J. Health Psychology.

[6] Radloff L.S. (1977). The CES-D scale: a self-report, depression scale for research in the general population. Appl. Psychol. Meas..

[7] Chon K.K., Choi S., Yang B. (2001). Integrated adaptation of CES-D in Korea. Korean J. Health Psychology.

[8] Spielberger C.D., Gorsuch R.L., Lushene R.E. (1970). Manual for the State-trait Anxiety Inventory.

[9] Kim J.T. (1978). The Relation between Trait Anxiety and Social Abilities: Focusing on Spielberger׳s STAI (Unpublished master׳s thesis).

[10] Taylor J.A. (1951). The relationship of anxiety to the conditioned eyelid response. J. Exp. Psychol..

[11] Han D.U., Lee C.H., Tak J.K. (1993). The validation of the Korean-version of Spielberger׳s STAI. J. Stud. Guidance.

